# Current practices in peripheral blood stem cell processing and cryopreservation: a nationwide survey of Korean transplant centers

**DOI:** 10.1007/s44313-025-00090-6

**Published:** 2025-07-22

**Authors:** Soo-Kyung Kim, Jaeeun Yoo, Jong-Han Lee, Ha-Eun Lee, Jae-Sook Ahn, Kyung-Nam Koh, Byung-Sik Cho, Seong-Kyu Park, Ho Joon Im, Hyunji Lee, Sun-Young Kong

**Affiliations:** 1 Department of Laboratory Medicine, Ewha Womans University College of Medicine, Seoul, Republic of Korea; 2 Department of Laboratory Medicine, Incheon St. Mary’s Hospital, Incheon, Republic of Korea; 3 Department of Laboratory Medicine, Yonsei University, Wonju College of Medicine, Wonju, Republic of Korea; 4 Research Institute of Metabolism and Inflammation, Yonsei University, Wonju College of Medicine, Wonju, Republic of Korea; 5 Targeted Therapy Branch, National Cancer Center, Goyang, Republic of Korea; 6 Department of Hematology-Oncology, Chonnam National University, Chonnam National University Hwasun Hospital, Hwasun, Jeollanamdo Republic of Korea; 7 Department of Pediatrics, Asan Medical Center Children’s Hospital, University of Ulsan College of Medicine, Seoul, Republic of Korea; 8 Department of Hematology, Catholic Hematology Hospital, Seoul St. Mary’s Hospital, College of Medicine, The Catholic University of Korea, Seoul, Republic of Korea; 9 Department of Internal Medicine, Soon Chun Hyang University College of Medicine, Seoul, Republic of Korea; 10 Department of Laboratory Medicine, Pusan National University School of Medicine, 20, Geumo-ro, Mulgeum-eup, Yangsan-si, Gyeongsangnam-do 50612, Republic of Korea; 11 Department of Laboratory Medicine, National Cancer Center, 323, Ilsan-ro, Ilsandong-gu, Goyang-si, Gyeonggi-do 10408 Republic of Korea

**Keywords:** Cell processing, Cryopreservation, Peripheral blood stem cells, Stem cell transplantation

## Abstract

**Purpose:**

Processing methods for hematopoietic stem cells vary significantly across institutions, with no standardized guidelines currently in place. This lack of standardization presents challenges in ensuring consistent quality and outcomes of stem cell transplantation procedures. This study investigated current practices in peripheral blood stem cell (PBSC) processing and storage among transplant centers in Korea to establish a foundation for the development of standardized guidelines.

**Methods:**

A comprehensive questionnaire was distributed to 46 hematopoietic stem cell transplantation centers in Korea, examining five key areas: PBSC collection procedures, use of cryopreservatives, cryopreservation protocols, quality control measures, and thawing protocols.

**Results:**

Analysis of the 29 responses revealed significant variations across different stages of PBSC handling. All centers used controlled-rate freezers, and 92.9% stored cells at temperatures below -150 ${}^\circ C$. However, other practices varied widely. Additional post-collection processing was performed by 53.8% of respondents. DMSO concentrations ranged from 5 to 15%, with diverse combinations of supplementary media. Notably, 28.6% of patients did not undergo post-thaw quality assessment tests.

**Conclusion:**

This study identified significant heterogeneity in PBSC processing practices across Korean transplant centers. These findings underscore the need for evidence-based standardized guidelines to ensure consistent product quality and improve transplantation outcomes.

**Supplementary Information:**

The online version contains supplementary material available at 10.1007/s44313-025-00090-6.

## Introduction

Recent advances in cellular therapies have led to the development of a wide range of therapeutic agents. The commercial and clinical success of these products invariably depends on the cryopreservation and storage of cellular materials. Optimization of cryopreservation is as crucial as refining cell culture processes to ensure maximum yield and product consistency.

Technical precision in cryopreservation is especially crucial in hematopoietic stem cell transplantation, where engraftment success largely depends on the number of viable CD34 + cells [1]. The cryopreservation of peripheral blood stem cells (PBSCs) involves multiple critical technical steps: cell harvesting, addition of cryopreservatives, freezing and storage, quality control, thawing, and infusion. Each of these steps requires strictly technical control to preserve cell viability and function [2]. Improper handling at any of stages may impair cellular function, highlighting the need for optimized protocols [3]. Therefore, maximizing the collection and preservation of viable CD34 + cells through well-controlled cryopreservation techniques is essential for the successful transplantation of PBSCs.

Although regulatory standards exist for hematopoietic stem processing exists, they only provide general recommendations without detailed procedural details [4]. This lack of standardization poses a major challenge to ensuring consistent product quality and clinical outcomes in stem cell transplantation procedures. Transplant centers often struggle to establish and implement intuition-specific protocols. To address this issue, we conducted a survey of institutions performing hematopoietic stem cell transplantation in the Republic of Korea. Our aim was to investigate the current practices in PBSC processing and storage, specifically examining variations in post-collection processing methods, cryopreservation protocols, quality control measures, and thawing procedures across institutions.

## Material and methods

We conducted a questionnaire survey to institutions involved in hematopoietic stem cell transplantation in Korea. An online survey link was distributed to 46 clinicians responsible for PBSC collection and cryopreservation at Korean transplantation centers between September and December 2024. The questionnaire covered five major aspects of stem cell handling: (1) PBSC collection procedures, (2) addition of cryopreservatives, (3) cryopreservation procedures (including container types, freezing methods, and storage conditions), (4) quality control measures (including viability testing and sterility assessment), and (5) thawing and infusion protocols. For each aspect, the participants were asked to provide detailed information about their current methodologies. The questionnaire also included an open-ended section where respondents could provide additional comments and feedback. The full questionnaire is provided in Supplementary Material. In cases where responses from the same institution showed inconsistencies between different questions, these were considered potential data entry errors and excluded from the final analysis. Additionally, responses indicating"don't know"or uncertain answers were also excluded from the corresponding analyses.

## Results

### Responsible departments and personnel

The analysis revealed significant variations across different stages of stem cell handling. Regarding the responsible department and personnel, the Department of Laboratory Medicine was primarily responsible for PBSC collections (75.9% of institutions), with nurses serving as the primary personnel involved (72.4%). Post-collection PBSC processing was also predominantly managed by the Department of Laboratory Medicine (65.5%), whereas the thawing procedures were most commonly conducted by the Department of Hemato-Oncology (62.1%) (Table 1).

**Table 1:** Departments and personnel responsible for each stage of peripheral blood stem cell handling

PBSC collection department	Laboratory medicine	22 (76%)
	Hemato-oncology	4 (14%)
	Laboratory medicine, Hemato-oncology	2 (7%)
	Nephrology	1 (3%)
PBSC collection personnel	Nurse	21 (72%)
	Medical technologist	3 (10%)
	Nurse, Medical technologist	3 (10%)
	Medical doctor, Nurse, Medical technologist	1 (3%)
	Medical doctor, Medical technologist	1 (3%)
PBSC processing department	Laboratory medicine	19 (66%)
	Hemato-oncology	5 (17%)
	Laboratory medicine, Hemato-oncology	1 (3%)
	Stem cell transplantation center	2 (7%)
	Hemato-oncology, Biological laboratory	1 (3%)
	Hemato-oncology, Pediatrics	1 (3%)
PBSC bag thawing department	Hemato-oncology	18 (62%)
	Laboratory medicine	6 (21%)
	Hemato-oncology, Pediatrics	3 (10%)
	Hemato-oncology, Biological laboratory	1 (3%)
	Stem cell transplantation center	1 (3%)

*Abbreviation*: *PBSC* peripheral blood stem cell

### Peripheral blood stem cell collection

For PBSC collection, 65.5% of institutions exclusively used the Spectra Optia (Terumo BCT, Lakewood, CO, USA), whereas 31.0% of used both Spectra Optia and Amicus (Fresenius Kabi, Bad Homburg, Germany) apheresis systems. The typical collection volume for adults ranged from 200 to 300 mL. In pediatric cases, some institutions reported varying collection volumes based on body weight, 56% of the centers either had no experience with or did not perform pediatric collections (Table 2).

**Table 2:** Peripheral blood stem cell collection devices and collection volumes

Collection device	Spectra Optia (Terumo BCT)	19 (65.5%)
	Spectra Optia (Terumo BCT), Amicus (Fresenius Kabi)	9 (31%)
	Amicus (Fresenius Kabi)	1 (3.4%)
Collection volume, adults (mL)	< 200	3 (11.1%)
	200–300^*^	20 (74.1%)
	≥ 300	4 (14.8%)
Collection volume, pediatrics (mL)	< 100	1 (4%)
	100–200	6 (24%)
	According to total blood volume	4 (16%)
	Not performed or no experience	14 (56%)

^*^Three institutions reported different collection volumes: allogeneic (250–280 mL) and autologous (100–220 mL) peripheral blood stem cell transplantation

### Post-collection processing

Post-collection processing varied considerably among institutions. A total of 53.8% reported performing additional plasma removal using centrifugation or cell processors, while 46.2% did not require additional processing. Post-collection processing methods included blood component centrifugation (34.6%), COBE 2991 (Terumo BCT; 15.4%), or a combination of both methods (3.8%).

For cryopreservation, institutions used various media combinations, most commonly including dimethyl sulfoxide (DMSO) with additives such as blood-derived components, cell culture media, buffered solutions, and anticoagulants. DMSO concentrations also varied between centers, with 36% of centers using 5–9% and 64% using 10–15%. All institutions used cryobags as cryopreservation containers, with bag volumes ranging from 50 to 500 mL. Most centers distributed the collected products across multiple bags, with the number of cryobags per collection ranging from 1 to > 5 (Table 3).

**Table 3:** Peripheral blood stem cell post-collection processing methods and cryopreservation container specifications

Post-collection processing equipment	Blood component centrifuge	9 (34.6%)
	COBE 2991 cell processor (Terumo BCT)	4 (15.4%)
	COBE 2991 cell processor (Terumo BCT), blood component centrifuge	1 (3.8%)
	Not performed	12 (46.2%)
Cryopreservation storage media	DMSO	2 (7.4%)
	DMSO + blood-derived components^*^	4 (14.8%)
	DMSO + cell culture media^†^	11 (40.7%)
	DMSO + buffered solutions^‡^	1 (3.7%)
	DMSO + blood-derived components + cell culture media	3 (11.1%)
	DMSO + blood-derived components + buffered solutions	1 (3.7%)
	DMSO + blood-derived components + anticoagulants^§^	1 (3.7%)
	DMSO + blood-derived components + cell culture media + anticoagulants	4 (14.8%)
DMSO concentration (%)	5–9	8 (36.4%)
	10–15	14 (63.6%)
Cryopreservation container	Cryo-bag	28 (100%)
	Cryo-vial	0 (0%)
Cryo-bag volume (mL)	50	5 (19.2%)
	60	1 (3.8%)
	100	2 (7.7%)
	250	16 (61.5%)
	500	2 (7.7%)
Number of cryo-bags used per single collection	1 bag	1 (4.3%)
	2–4 bags	20 (87%)
	≥ 5 bags	2 (8.7%)

^*^Blood-derived components: albumin and autoplasma

^†^Cell culture media: RPMI1640, medium199, Iscove's modified Dulbecco's medium (IMDM), minimum essential medium (MEM)

^‡^Buffered solutions: HBSS (Hank's balanced salt solution), normal saline

^§^Anticoagulants: heparin

*Abbreviation*: *DMSO* dimethyl sulfoxide

### Cryopreservation

All institutions used controlled-rate freezers for cryopreservation. While 92.9% of centers stored cells below −150 ℃, 7.1% maintained storage below −80 ℃. The liquid nitrogen containers used were predominantly from LABS series (Korea Cryogenics, Seongnam, Korea) or MVE series (MVE Biological, Ball Ground, GA, USA). Storage duration policies for cryopreserved PBSC varied widely: 17.9% of institutions reported a storage policy of 5–9 years, and another 17.9% reported storage exceeding 10 years. Notably, 53.6% of institutions reported having no specific policy regarding storage duration (Table 4).

**Table 4:** Peripheral blood stem cell cryopreservation methods and storage policies

Cryopreservation method	Use controlled rate freezer	28 (100%)
Cryopreservation temperature	< −150 ℃ < −80 ℃	26 (92.9%)2 (7.1%)
Cryopreservation equipment	LABS series (Korea cryogenics)	14 (50%)
	MVE series (MVE Biological Solution)	10 (35.7%)
	MVE series (MVE Biological Solution), LABS series (Korea cryogenics)	2 (7.1%)
	VIVI series (Bis)	2 (7.1%)
Storage duration regulation for cryopreserved PBSC products	Established policy	13 (46.4%)
	< 1 year	1 (3.6%)
	1–4 years	2 (7.1%)
	5–9 year	5 (17.9%)
	≥ 10 years	5 (17.9%)
	No established policy	15 (53.6%)

*Abbreviation*: *PBSC* peripheral blood stem cell

### Post-thaw quality assessment

Post-thaw quality assessment practices varied as follows: 50% performed cell viability testing, 21% conducted CD34 + cell counts, 18% performed sterility testing, and 29% did not. Among the institutions that performed viability testing, most used trypan blue staining (13/15, 84.6%) (Table 5).

**Table 5:** Post-thaw quality control tests and viability assessment methods for peripheral blood stem cell products

Post-thaw quality assessment tests	Cell viability	9 (32.1%)
	CD34 cell count	2 (7.1%)
	Sterility test	3 (10.7%)
	Cell viability, CD34 cell count	1 (3.6%)
	Cell viability, CD34 cell count, sterility test	1 (3.6%)
	Cell viability, TNC count	1 (3.6%)
	Cell viability, CD34 cell count, TNC count	1 (3.6%)
	Cell viability, TNC count, TNC recovery rate	1 (3.6%)
	CD34 cell count, TNC count, TNC recovery rate, CD34 recovery rate, sterility test	1 (3.6%)
	Not performed	8 (28.6%)
Post-thaw cell viability test	Trypan blue dye staining	13 (48.1%)
	7-AAD viable CD34 + cell flowcytometry	2 (7.4%)
	Not performed	12 (44.4%)

*Abbreviations 7-AAD* 7-Aminoactinomycin D, *PBSC* peripheral blood stem cell, *TNC* total nucleated cells

### Thawing

Most institutions (78.6%) performed PBSC thawing at the patients’ bedside using the water bath method. For pre-infusion processing, 11.1% of the centers reported using centrifugation for supernatant removal. Although 88.9% of the institutions did not perform additional processing before infusion, several centers reported that they restricted the number of bags infused simultaneously to minimize DMSO exposure (Table 6).

**Table 6:** Peripheral blood stem cell thawing procedures and pre-infusion processing methods

PBSC product thawing location	Bedside	22 (78.6%)
	Aseptic ward	3 (10.7%)
	Stem cell collection center	3 (10.7%)
Thawing method for PBSC bags	37 ${}^\circ C$ water bath	27 (96.4%)
	37 ${}^\circ C$ incubator	1 (3.6%)
Pre-infusion procedures for DMSO toxicity reduction	Supernatant removal after centrifugation^*^	3 (11.1%)
	Not performed	24 (88.9%)

^*^One center performed centrifugation only in cases of renal dysfunction

*Abbreviations*: *DMSO* dimethyl sulfoxide, *PBSC* peripheral blood stem cell

## Discussion

There were significant variations in PBSC handling across the transplantation centers. Notable differences include post-collection processing methods, DMSO concentrations, and storage duration policies, with many centers lacking established guidelines. In addition, quality control measures varied, with a substantial number of centers conducting no post-thaw quality assessment tests.

The survey revealed considerable variations in the involvement of department-responsible personnel across institutions for PBSC handling. While the Department of Laboratory Medicine predominantly managed the collection (75.9%) and processing (65.5%), thawing procedures were mainly conducted in the Hemato-Oncology Department (62.1%).

Most institutions used Spectra Optia for PBSC collection, with some centers maintaining both the Spectra Optia and Amicus apheresis systems. The collection volumes were relatively standardized for adult patients, with 74.1% of the centers collecting 200–300 mL. 56% of the centers reported no experience or did not perform pediatric collections.

There was significant heterogeneity in the post-PBSC collection processing and cryopreservation practices. While 53.8% of the institutions performed additional processing using either cell processors or centrifuges, 46.2% did not conduct post-collection processing, highlighting a fundamental difference in the approaches. The composition of cryopreservation media showed notable variation, with most centers using dimethyl sulfoxide (DMSO) in cell culture media (40.7%), whereas others employed various combinations of blood-derived components, buffered solutions, and anticoagulants. DMSO concentrations also varied, with 63.6% of centers using 10–15% concentration versus 36.4% using 5–9% concentration.

Stem cell preservation requires cryoprotective agents to prevent cellular damage caused by ice crystal formation. DMSO is the primary cryoprotectant used to prevent intracellular ice formation and preserve cell viability [5–7]. However, DMSO can cause dose-dependent adverse effects, ranging from mild symptoms such as nausea and hypotension to severe complications including cardiac arrhythmias and neurological effects [8]. Optimization of the DMSO concentration is crucial, with a previous study indicating that 5% DMSO combined with pentastarch can effectively balance reduced toxicity and maintain cell viability [6]. In contrast, many centers continue to use 7.5–10% DMSO for its proven protective effects, often implementing post-thaw washing to reduce toxicity [8–10].

DMSO is often combined with supplementary solutions to enhance preservation and reduce toxicity. Although albumin and saline have demonstrated benefits in membrane protection and toxicity reduction, the evidence of specific culture media benefits remains limited [10, 11]. Our survey revealed that institutions used various combinations of blood-derived components, buffered solutions, and cell culture media, including RPMI 1640, Medium 199, Iscove's modified Dulbecco's medium (IMDM), and minimum essential medium (MEM).

All the institutions used cryobags for PBSC cryopreservation. The volume of cryobags varied among centers, with 250 mL being the most commonly used volume. Overall, bag volumes ranged from 50 to 500 mL. Regarding the number of bags used per collection, most institutions divided the collected product into to 2–4 bags for banking. According to the FACT-JACIE International Standards (8th Edition, 2021) and AABB-ISCT Joint Working Group Report (2022), the total blood volume processed per session typically ranges from 10 to 24 L, resulting in a final PBSC product volume of 150 to 500 mL [4, 12]. To enhance freezing efficiency and reduce DMSO toxicity, PBSCs are not stored in a single 500 mL bag, but are instead divided into multiple smaller bags, typically containing 100–200 mL each. FACT-JACIE recommends storing PBSCs in 50–200 mL aliquots, whereas AABB-ISCT reports that most centers use 100–250 mL per cryopreservation bag.

Controlled-rate freezing remains the standard method for PBSC cryopreservation, and our survey confirmed that all participating institutions consistently employed this method. Controlled-rate freezing is essential because the heat released at the transition point (4 °C), when water molecules begin to form structured ice-crystals, can damage stem cells. The process employs a two-phase cooling approach: an initial slow rate (1–2 °C/minute) to −40 °C, followed by faster cooling (3–5 °C/minute) to −120 °C, optimizing stem cell preservation.

Hematopoietic stem cell cryopreservation temperatures have been categorized into three main ranges: −196 ℃ (liquid phase nitrogen), −156 ℃ (vapor phase nitrogen), or −80 ℃ (mechanical freezer) nitrogen [5]. Current evidence indicates that long-term PBSC storage is best maintained at temperatures below −150 °C, as storage at −80 ℃ may compromise cell viability [13]. Our survey showed that 92.9% of institutions maintained PBSC storage at temperatures below −150 °C, while only 7% stored at −80 °C.

Our survey revealed significant variations in PBSC storage duration policies. A total of 46.4% of institutions had storage duration guidelines, whereas 53.6% had no established guidelines. Among those with established policies, the storage duration varied considerably: 3.6% stored PBSC for less than one year, 7.1% for 1–5 years, and 35.7% for over five years. Research demonstrates that PBSC stored below −150 ℃ can maintain viability and engraftment capacity beyond 10 years [14–16]. However, rigorous temperature monitoring and systematic quality-assessment protocols are required to ensure long-term storage [2].

Post-thaw quality assessment practices vary considerably across institutions. Viability testing is essential for ensuring the quality and functionality of cryopreserved PBSC. Among the institutions that conducted viability testing, trypan blue dye staining was the predominant method (48.1%), with only a small proportion (7.4%) using the more sophisticated 7-Aminoactinomycin D (7-AAD) viable CD34 + cell flow cytometry. Trypan blue staining is simple and cost-effective but lacks functional assessment, whereas flow cytometry, such as the 7-AAD viable CD34 + cell assay, offers precise evaluations but requires specialized equipment and trained personnel. Other centers employ various combinations of tests, including total nucleated cell (TNC) counts, recovery rates, and sterility testing. Notably, 44.4% of institutions did not perform any cell viability testing and 28.6% of centers reported conducting no post-thaw quality assessment tests of any kind. The wide variation in quality assessment practices and the high proportion of centers that do not perform viability testing suggest the critical need to establish minimum quality control standards for post-thaw PBSC products.

Most institutions (78.6%) performed PBSC thawing at the patients’ bedsides. Almost all centers (96.4%) used 37 ℃ water bath method. PBSC thawing using 37 ℃ water bath until complete ice crystal dissolution is considered as standard protocol [17].

Post-thaw DMSO removal was not performed in 88.5% of institutions. Some studies have reported limiting the number of bags infused simultaneously as an alternative approach for reducing DMSO exposure. Although DMSO removal primarily benefits patients by reducing toxicity [18], the procedure is time-consuming. Recent evidence suggesting greater stem cell tolerance to DMSO exposure [19] has led to a debate regarding its necessity.

In the survey comments, respondents consistently highlighted the operational challenges arising from the lack of standardized guidelines. Most institutions expressed difficulties in establishing their protocols, particularly for cryopreservation medium composition and cell-processing procedures. The respondents emphasized a strong desire for standardized protocols and regular training programs, further underlining the need for comprehensive and practical guidelines.

Our nationwide survey revealed significant heterogeneity in PBSC processing and cryopreservation practices across transplant centers in Korea. While some practices were consistent, such as the universal use of controlled-rate freezing and predominant storage below −150 °C, other critical areas showed considerable variation. These findings highlight the need for standardized guidelines for PBSC processing and storage, particularly regarding quality control measures and storage policies.

## Conclusion

Despite the critical importance of stem cell processing and storage for transplantation outcomes, our study revealed substantial heterogeneity in practices across institutions. The variations observed in the processing methods, cryopreservation, and storage policies highlight the urgent need for evidence-based standardized guidelines. While this study provides a comprehensive overview of the current practices in Korea, further research is needed to establish optimal protocols. Developing standardized guidelines is crucial for ensuring consistent product quality, reducing institutional variability, and ultimately improving patient outcomes of hematopoietic stem cell transplantation.

## Supplementary Information

Supplementary Material 1

## Data Availability

No datasets were generated or analysed during the current study.
