# Supplementary material for: Current practices in peripheral blood stem cell processing and cryopreservation: a nationwide survey of Korean transplant centers
Source: Blood Res. 2025 Jul 22;60(1):41. doi: 10.1007/s44313-025-00090-6 (PMC12283502; doi:10.1007/s44313-025-00090-6)
Supplement: Supplementary file 1 — Supplementary Material 1 [file 44313_2025_90_MOESM1_ESM.docx]

Supplementary Material

Survey Questionnaire on PBSC Processing and Cryopreservation Practices

*The following questions concern general characteristics. Please check (✓) the appropriate box or fill in directly. Please answer all questions completely.*

1. Please provide your institution name. _______________
2. Please specify your department. _______________
3. Please select the type of your medical institution.
   1. Tertiary hospital
   2. General hospital
   3. Hospital
   4. Other: _______________
4. In which region is your medical institution located?
   1. Gangwon
   2. Gyeonggi
   3. Gyeongnam
   4. Gyeongbuk
   5. Gwangju
   6. Daegu
   7. Daejeon
   8. Busan
   9. Seoul
   10. Sejong
   11. Ulsan
   12. Incheon
   13. Jeonnam
   14. Jeonbuk
   15. Jeju
   16. Chungnam
   17. Chungbuk
5. How many beds does your medical institution have?
   1. Less than 500 beds
   2. 500-999 beds
   3. 1000-1499 beds
   4. 1500 beds or more

The following questions concern peripheral blood stem cell collection. Please check (✓) the appropriate box or fill in directly. Please answer all questions completely.

1. Which department is responsible for PBSC collection? (This refers only to collection, excluding processing, freezing, and storage) (Select all that apply)
   1. Laboratory medicine
   2. Hematology-oncology
   3. Pediatrics
   4. Other: _______________
2. Who is responsible for PBSC collection? (This refers only to collection, excluding processing, freezing, and storage) (Select all that apply)
   1. Nurse
   2. Medical technologist
   3. Other: _______________
3. What equipment do you use for PBSC collection? (Select all that apply)
   1. Spectra Optia
   2. Amicus
   3. Other: _______________
4. What is the approximate collection volume for PBSC collection?

1) Adults: _______ mL

2) Pediatrics: _______ mL

The following questions concern peripheral blood stem cell cryopreservation processing. Please check (✓) the appropriate box or fill in directly. Please answer all questions completely.

1. Which department is responsible for PBSC processing (preprocessing, freezing, storage, etc.)? (Select all that apply)
   1. Laboratory medicine
   2. Hematology-oncology
   3. Pediatrics
   4. Other: _______________
2. Do you perform cell washing, plasma removal, or cell concentration after peripheral blood stem cell collection?
   1. Use COBE 2991 cell processor
   2. Use blood component centrifuge
   3. Do not perform cell washing, removal, or concentration
   4. Other: _______________
3. What type of container do you use to store peripheral blood stem cells?
   1. Cryo-Bag
   2. Cryo-Vial
   3. Other: _______________
4. What is the volume of the Cryo-Bag or Cryo-Vial you use?
   1. 50 mL
   2. 250 mL
   3. 500 mL
   4. 750 mL
   5. Other: _______________
5. How many freezing bags do you use for a single collection from one donor?
   1. 2 bags
   2. 3 bags
   3. 4 bags
   4. Other: _______________
6. What agents do you use as cryoprotectants? (Select all that apply)
   1. DMSO
   2. Glycerol
   3. Hydroxyethyl starch
   4. Trehalose
   5. Albumin
   6. Dextran
   7. Autologous plasma
   8. Fetal bovine serum
   9. Other: _______________
7. Please describe the types and volumes of media and cryoprotectants used for PBSC cryopreservation as shown in the example below. This is the most variable aspect among institutions. Please provide detailed information.

**Example:**

| Component (Product name) | | Volume (mL) |
| --- | --- | --- |
| Medium | RPMI 1640 (Gibco Laboratories) | 36 |
|  | DMSO (OriGen Biomedical CryoPur) | 12 |
|  | Autologous plasma | 12 |
| PBSC | | 60 |
| Other 1: | |  |
| Other 2: | |  |
| Total volume per freezing bag | | 120 |

**Your institution: Target population Adults**

| Component (Product name) | | Volume (mL) |
| --- | --- | --- |
| Medium |  |  |
|  |  |  |
|  |  |  |
| PBSC | |  |
| Other 1: | |  |
| Other 2: | |  |
| Total volume per freezing bag | |  |

**For pediatric patients (if different)**

| Component (Product name) | | Volume (mL) |
| --- | --- | --- |
| Medium |  |  |
|  |  |  |
|  |  |  |
| PBSC | |  |
| Other 1: | |  |
| Other 2: | |  |
| Total volume per freezing bag | |  |

1. What is the final DMSO concentration?
   1. 10%
   2. 5% or more, less than 10%
   3. Less than 5%
   4. Not used
   5. Other: _______________

The following questions concern peripheral blood stem cell storage. Please check (✓) the appropriate box or fill in directly. Please answer all questions completely.

1. Regarding PBSC freezing rate, please select the applicable option.
   1. Use controlled rate freezer
   2. Do not use controlled rate freezer
   3. Other: _______________
2. What is the PBSC storage temperature?
   1. Below -150℃
   2. Below -80℃
   3. Other: _______________
3. What equipment do you use for PBSC storage?
   1. MVE series liquid nitrogen storage container
   2. Korea Cryogenics KC series liquid nitrogen storage container
   3. Deep freezer
   4. Other: _______________
4. Do you have institutional regulations regarding PBSC storage duration? If yes, what is the storage period?
   1. No regulations
   2. Established policy: Less than 1 year
   3. Established policy: 1-5 years
   4. Established policy: 5-10 years
   5. Established policy: 10 years or more
   6. Established policy: Permanent storage
   7. Other: _______________

The following questions concern quality control of peripheral blood stem cells. Please check (✓) the appropriate box or fill in directly. Please answer all questions completely.

1. What tests do you perform after thawing PBSC products? (Select all that apply)
   1. Total nucleated cell (TNC) count
   2. CD34 cell count
   3. Cell viability
   4. TNC recovery rate
   5. CD34 recovery rate
   6. Colony forming unit (CFU) assay
   7. Sterility test (culture, etc.)
   8. No testing performed
   9. Other: _______________
2. Do you perform cell viability testing after thawing PBSC products? What tests do you perform? (Select all that apply)
   1. Trypan blue staining
   2. 7-AAD viable total nucleated cell flow cytometry
   3. 7-AAD viable CD34+ cell flow cytometry
   4. CBC analyzer-based testing
   5. No cell viability testing performed
   6. Other: _______________
3. Do you have regulations for periodic stability assessment of long-term stored PBSC products?
   1. Yes
   2. No
   3. Other: _______________

The following questions concern pre-transplant processing of peripheral blood stem cells. Please check (✓) the appropriate box or fill in directly. Please answer all questions completely.

1. Do you perform procedures to reduce DMSO toxicity before patient infusion? If yes, what methods do you use?
   1. Supernatant removal after centrifugation
   2. Not performed
   3. Other: _______________
2. Which department is responsible for thawing frozen PBSC bags?
   1. Laboratory medicine
   2. Hematology-oncology
   3. Pediatrics
   4. Other: _______________
3. Where do you thaw frozen PBSC bags?
   1. Patient bedside
   2. Stem cell collection room
   3. Other: _______________
4. How do you thaw frozen PBSC bags before infusion?
   1. 37℃ water bath
   2. 37℃ incubator
   3. Other: _______________

The following section is for additional suggestions. Please provide written responses. Please fill in directly.

1. Please feel free to describe any additional comments you would like to make regarding PBSC. ________________________________
